# A Modified Distributed Bees Algorithm for Multi-Sensor Task Allocation †

**DOI:** 10.3390/s18030759

**Published:** 2018-03-02

**Authors:** Itshak Tkach, Aleksandar Jevtić, Shimon Y. Nof, Yael Edan

**Affiliations:** 1 Department of Industrial Engineering and Management, Ben-Gurion University of the Negev, 8410501 Beer-Sheva, Israel; yael@bgu.ac.il; 2 Institut de Robòtica i Informàtica Industrial, CSIC-UPC, 08028 Barcelona, Spain; ajevtic@iri.upc.edu; 3 PRISM Center and School of Industrial Engineering, Purdue University, West Lafayette, IN 47907, USA; nof@purdue.edu

**Keywords:** multi-agent systems, distributed task allocation, swarm intelligence, sensor deployment

## Abstract

Multi-sensor systems can play an important role in monitoring tasks and detecting targets. However, real-time allocation of heterogeneous sensors to dynamic targets/tasks that are unknown a priori in their locations and priorities is a challenge. This paper presents a Modified Distributed Bees Algorithm (MDBA) that is developed to allocate stationary heterogeneous sensors to upcoming unknown tasks using a decentralized, swarm intelligence approach to minimize the task detection times. Sensors are allocated to tasks based on sensors’ performance, tasks’ priorities, and the distances of the sensors from the locations where the tasks are being executed. The algorithm was compared to a Distributed Bees Algorithm (DBA), a Bees System, and two common multi-sensor algorithms, market-based and greedy-based algorithms, which were fitted for the specific task. Simulation analyses revealed that MDBA achieved statistically significant improved performance by 7% with respect to DBA as the second-best algorithm, and by 19% with respect to Greedy algorithm, which was the worst, thus indicating its fitness to provide solutions for heterogeneous multi-sensor systems.

## 1. Introduction

Distributed multi-sensor systems can play an important role in monitoring and detection applications, due to their capability to cover the entire area and ensure a robust response to dynamic situations [1,2,3]. Fusion of various sensor inputs (e.g., visible, infrared, microwave, and acoustic) can further increase performance and reliability [4] since each sensor has different capabilities (e.g., detection distance, angle, resolution). Most multi-sensor monitoring systems rely on a very large number of sensors, usually swarms, to cover the entire area and to be able to allocate tasks [5,6], which makes the problem of the efficient allocation of sensors to tasks NP-hard [6]. When there are several tasks that require the same sensors, a decision must be made regarding which sensor to allocate to which task. In complex manufacturing systems, this problem becomes more complicated as tasks arrive at different locations and unknown times, and sensors are heterogeneous. The allocation depends on the sensors’ availability and performance, and the priorities of individual tasks.

This research focuses on allocation of stationary heterogeneous sensors to dynamic tasks. The sensors’ locations are a priori set and are tested for different distribution strategies. The tasks appear randomly with a uniform distribution. The task allocation is performed by adapting a more recent Modified Distributed Bees Algorithm (MDBA) [7]. The current paper details the problem formulation and provides an in-depth and complete analysis of the results. Evaluations include comparison to another biologically inspired Bees System (BS) algorithm [8], systems’ scalability for the number of sensors and tasks, and the impact of the control parameters on task allocation. The new results include systems’ performance based on sensors’ distribution and the number of sensors that are used, and the number of treated tasks by each sensor in each algorithm.

In this paper there is no limitation in the number of sensors that can be allocated to a single task. This eliminates the conflict of demanding the same task by multiple sensors.

MDBA’s performance was compared to four state-of-the-art algorithms that were adjusted for the specific application. The choice of algorithms was made to include different approaches; specifically, we compared greedy vs. heuristic and swarm-based vs. market-based approaches. Accordingly, the algorithms compared were DBA, market-based algorithm [9], greedy algorithm [10], and Bees System. Due to the complexity of the problem a comparison was made with decentralized versions of market-based and greedy algorithms. The centralized version of these algorithms was implemented before in [7] but yielded poor results.

The remainder of this paper is organized as follows. Section 2 describes the related work. Section 3 defines the Multi-Sensor Task Allocation problem. Section 4 describes the algorithms formulation followed by Section 5, which presents the simulation setup. Section 6 presents the simulation results, with conclusions being presented in Section 7.

## 2. Related Work

In multi-sensor multi-task applications, the problem of task allocation refers to selection of suitable sensor combinations to be allocated to perform different tasks [11]. Many known allocation strategies require tasks to be defined a priori so that they can be allocated efficiently to specific sensors [4] and robots [12,13,14]. However, in real-world scenarios, the upcoming tasks are usually undefined and have a dynamic nature (e.g., unknown arrival times, change in location, different priorities [15]). Therefore, it is necessary to decide when and where to apply sensors to ensure maximum task attendance, and to decide how to reallocate sensors to adapt to the dynamic tasks locations, arrival times, and priorities, while minimizing the number of the required sensors [11]. For the allocation problem, each individual sensor is considered as an agent with particular capabilities [16]. Sensor allocation has been previously achieved using different methods, such as a network flow optimization model and cause effect graph [4,17], data association algorithm in sensor network [18], market-based approaches applied in multi agent systems [19,20,21,22,23], and dynamic allocation and coalition-based approach [24,25,26,27,28,29,30,31].

Swarm intelligence, inspired by the emergent behavior of social insects, such as ants, bees, and termites, flocks of birds, and schools of fish, has been used to model the behavior of intelligent multi-agent systems [32,33,34,35,36,37]. In swarm intelligence algorithms, cooperating agents interact by exchanging locally available information, such that the global objective is obtained more efficiently than it would be done by agents that perform tasks individually [38]. Swarms can be useful because they can deliver performance that is better than the sum of the parts. These algorithms have been applied to applications of multi-agent exploration and path formation [39], energy optimization in sensor networks [40], multi-site deployment [41], parallel computing optimization [42], cooperative transport and vehicle routing [43,44], feature selection [45], intruder detection [46], resource allocation [47], multi-robot task allocation and tracking applications [48,49], and so forth. Disadvantages of swarm intelligence may include needless activities of the agents, conflicts, and slow global response to a change in the environment [38,50].

The Distributed Bees Algorithm (DBA) is a swarm intelligence algorithm that is suitable for implementation in a multi-agent system and is also scalable with low computational overhead [48]. This algorithm uses a bottom-up design topology that makes the multi-agent systems autonomous, scalable, robust, and adaptive to changes in their environment. Initially, DBA was proposed to solve the problem of homogeneous agents. By neglecting the agent ability-based performance parameter, it can be applied also to heterogeneous multi-agent systems (without addressing the different ability of each agent). But, the fact that it models the system as a swarm of agents with equal abilities limits the overall system performance. The first implementation of DBA to heterogeneous agents was developed in [7].

Both, DBA and MDBA use swarm intelligence principles to provide distributed multi-sensor system control. The MDBA algorithm introduces sensor’s performance as an additional control parameter for sensor allocation. In previous work, DBA was applied to a homogeneous mobile sensor network and stationary tasks, without the reallocation of sensors during task execution [48]. In this paper, the DBA’s parameters were set to allocate dynamic tasks to a heterogeneous multi-sensor system. Once a sensor finds a task, it informs the neighboring sensors about the found task and its parameters, using broadcast communication (as in [10,51,52,53]). This message is then forwarded by these sensors over the entire network, as in [53]. From that moment, the sensors are aware of the detected task. Even though the sensors use broadcast communication to share the estimated location and priority of the tasks, task allocation is performed in a decentralized manner, where each sensor makes an autonomous decision that is based on the information that it has received.

Market-based algorithms apply an auctioning mechanism and the principles of market economy to allocate tasks in a multi-agent system [54]. In this virtual economy, the sensors are traders, tasks are traded commodities, and virtual money acts as currency. Sensors compete to win tasks by participating in auctions in which each sensor acts to maximize its individual profit and simultaneously improve the efficiency of the team. The limitations of market-based algorithms include a limited ability to deal with unknown and dynamic situations, and high demand of communication and computation resources [55].

The greedy algorithm is a heuristic algorithm widely applied to optimization problems [56]. It makes a locally optimal choice at each stage aiming to find a global optimum, but fails if the locally optimal solutions are not part of the global optimum [57]. Therefore, for large scale search problems, it has a tendency of getting stuck in a local optimum.

A comprehensive comparison of related work on agent management and coordination has been published in [58]. The novelty of the proposed approach is that it enables the efficient allocation of a heterogeneous swarm of sensors to dynamic tasks by a decentralized coordination that does not rely on a central agent (which can be a single point of failure) to coordinate sensors. The proposed approach is also scalable in terms of different number of tasks and sensors.

## 3. Problem Definition

**Figure 1:**
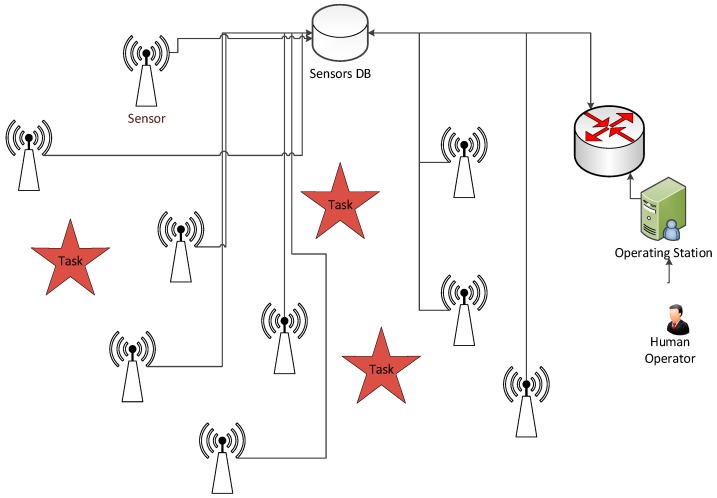
A monitoring sensor network with sensors, tasks and sensors DB that informs operators about the completed tasks, but does not contribute to the task allocation algorithm.

The problem deals with real-time allocation of stationary heterogeneous sensors to unknown tasks arriving at unknown times and locations. The tasks’ occurrence is dynamic and unpredictable with different levels of importance of each task (defined as priority). Examples of such tasks include surveillance (gathering information on desired objects [59]), security monitoring (preventing theft of goods and threats [60]), fire monitoring (forest fire detection and protection [61]), among many others. The sensors must be allocated to the tasks as fast as possible. The system consists of multiple sensors that are capable of performing each task with different performances (Figure 1). Sensory performance is defined a priori based on the sensor’s features, namely detection distance, resolution, and response time. Each sensor can only be allocated to one task at any given time and can be reallocated to another task at any moment. The priority of a task is an application-specific scalar value, where a higher priority value represents a task that has higher importance and must be attended to faster than other tasks. Higher priority tasks also have a higher benefit for completing them. The goal is to allocate each sensor an appropriate task at an appropriate time and to ensure that all tasks are completed in minimal time.

When there are several tasks that require the same sensors, the allocation depends on the sensors’ availability and performance, the physical distance of sensors from the tasks and the priorities of individual tasks. The following assumptions were considered in the proposed scenario, similar to research performed in [48]:

- the tasks occurrence is unknown a priori;
- all of the tasks within a sensor’s range can be allocated to that sensor;
- decision-making for each sensor takes place as soon as a new task is introduced;
- sensors can be reallocated to another task during execution. An abandoned task keeps its remaining execution time, until a new sensor is allocated to it. Following [59] the system is defined with the following characteristics:
- sensors are stationary; and,
- tasks remain stationary after their occurrence.

Based on taxonomy proposed by [62], the problem of allocating sensors to tasks corresponds to a localization and observation problem. The task localization involves several sensors and it is most often a multi-sensor problem to improve knowledge about the task involving selection of different viewpoints to maximize the information gain. The observation problem involves several sensors and several tasks in order to maximize the number of observed tasks and to minimize the time during which any task is not observed by at least one of the sensors.

A system objective function was developed to allow computation of the expected value of system performance given parameters of the sensors and tasks. System performance is defined as the collective performance of sensors, task priorities values and distances of sensors from tasks. Consider a population of *N* sensors to be allocated among *M* tasks. We denote the collective performance of the system by *V_I_*, a nonnegative integer, calculated as:

(1) $$V_{I}=\max\{S+\varphi H\},0<\varphi <1$$

(2) $$S=\sum_{i=1}^{M}\sum_{k=1}^{N}V_{ik}\cdot \frac{1}{D_{ik}}$$

(3) $$H=\sum_{j=1}^{M}F_{j},\;j\in completed\;tasks$$

(4) $$V_{ik}=0\;if\;k\text{-}th\;sensor\;is\;not\;assigned\;to\;task\;i$$

where *S* is the collective performance of the sensors, *V_ik_* is the *k*-th sensor’s performance on the *i*-th task, and *N* is the number of sensors in the system. *H* is the sum of the priorities of tasks in the system that were successfully completed and *φ* is a bias parameter for the importance of *H* relative to *S*. *F_j_* is the priority of *j*-th task and *M* is the total number of tasks. These values were pre-defined in the simulation setup based on [56].

**Figure 2:**
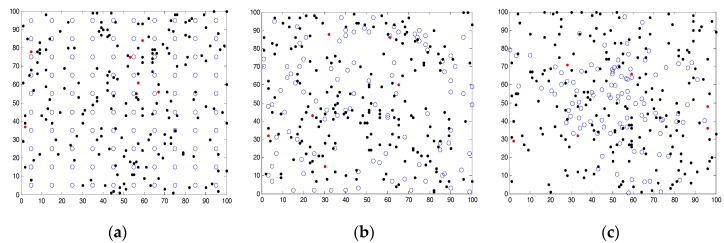
Simulation map of the arena with three sensors distributions—(**a**) grid distribution; (**b**) uniformly distributed random deployment; (**c**) normally distributed random deployment; deployed sensors are marked as blue circles, incomplete tasks are marked by red dots, and completed tasks are marked by black dots.

Sensors are distributed in the arena with a particular distribution strategy (as in Figure 2) and can be allocated to tasks within their detection range. The sensors are stationary. One of the parameters that affect system performance is the distance of the sensors from tasks. The performance degrades as the distance increases, due to the degradation of sensors recognition capabilities.

The Euclidean distance between the sensor and the task in a two-dimensional arena is given by:

(5) $$D_{ik}=\sqrt{{\left(x_{i}-x_{k}\right)}^{2}+{\left(y_{i}-y_{k}\right)}^{2}}$$

where (*x_i_*, *y_i_*) and (*x_k_*, *y_k_*) represent task’s and sensor’s coordinates in the arena, respectively.

Let *F*ϵ{*F_1_*, …, *F_M_*} denote the set of normalized priorities of the available tasks in the queue. Normalized priorities are calculated as fractions of the sum of priorities of all available tasks:

(6) $$F_{i}=\frac{f_{i}}{\sum_{j=1}^{M}f_{j}}$$

where *f_i_* is a priority of the task *i*.

Each task has a time limit, or a deadline:

(7) $$\Delta_{i}=\frac{1}{F_{i}}>0,F_{i}>0$$

where *Δ_i_* decreases with time till it reaches a value of 0 which indicates that the task *i* is not relevant any more.

Once a sensor is allocated to a task, it monitors this particular task (with a performance *V_ik_*). A task is considered to be completed once sensors are allocated to it. 

Each task is assumed to have an initial completion time value *t_init_*. The task execution time value is modeled as a function of the initial completion time that is required for the task and the performances of sensors that are allocated to it. This dynamic value is continuously updated (Equation (8)).

(8) $$t_{ex_{i}}=\{\begin{array}{ll} t_{init}\frac{1}{V_{i}}-t_{p_{i}} & \;\mathrm{if}\;V_{i}>0,\Delta_{i}>0\\ t_{init} & \;\mathrm{if}\;V_{i}=0,\Delta_{i}>0 \end{array}$$

where *t_exi_* is the remaining execution time of task *i* and *t_pi_* is the elapsed time of task *i* execution. When *t_exi_* reaches 0 value, the task is completed, and it is removed from the arena by the broadcast communication.

When new tasks are announced, steps (1)–(8) are updated respectively. The overall task completion time *T* is defined as a sum of the individual tasks completion times *t_ci_*:

(9) $$T=\sum_{i=1}^{M}t_{c_{i}}$$

(10) $$t_{c_{i}}=t_{i_{f\_completion}}-t_{i_{arrival}},\Delta_{i}>0$$

The task completion time is derived by the amount of time elapsed from its arrival *t_iarrival_* to the full completion *t_if_completion_*.

## 4. Algorithms

The developed MDBA [7] is compared to the original DBA [48], BS [8], a greedy algorithm [10], and to a market-based algorithm [9], which were implemented to fit the sensor allocation problem, as described below.

### 4.1. Distributed Bees Algorithm

In this algorithm, each sensor is represented as a ‘bee’, and sensor utility, *p_ik_*, is defined as a probability that the sensor *k* is allocated to the task *i* and depends on both priority and the distance of the task from the sensor:

(11) $$p_{ik}=\frac{F_{i}^{\alpha }{\left(\frac{1}{D_{ik}}\right)}^{\beta }}{\sum_{j=1}^{M}F_{j}^{\alpha }{\left(\frac{1}{D_{jk}}\right)}^{\beta }}if\Delta_{i}>0$$

where *α* and *β* are control parameters that bias importance of the priority and distance, respectively, (*α*, *β* > 0; *α*, *β ϵ R*). The probabilities *p_ik_* are normalized, and it is easy to show that:

(12) $$\sum_{i=1}^{M}p_{ik}=1$$

The DBA decision-making mechanism uses a wheel-selection rule used in [48], where each sensor is allocated a task from a set of available tasks based on its probability.

### 4.2. Modified Distributed Bees Algorithm

In the MDBA, the original DBA sensor utility function is modified to take advantage of heterogeneous sensors with different performances aiming to improve system performance by correlating the sensors’ utility with their performances.

To apply MDBA to heterogeneous sensors, a control parameter was defined in [7] as a function of the sensor’s performance on a task. When a sensor receives information about an available task it calculates its performance for that task. The sensor’s utility function is updated accordingly, and depends on the task priority, the distance from the task and the sensor’s performance on that task:

(13) $$p_{ik}=\frac{F_{i}^{\alpha }{\left(\frac{1}{D_{ik}}\right)}^{\beta }V_{ik}^{\chi }}{\sum_{j=1}^{M}F_{j}^{\alpha }{\left(\frac{1}{D_{jk}}\right)}^{\beta }V_{jk}^{\chi }}if\Delta_{i}>0$$

where *γ* is a control parameter that biases the importance of the sensors performance and *V_ik_* is the performance of sensor *k* on task *i*.

The MDBA decision-making mechanism applies the same wheel-selection rule that is used in DBA to choose from a set of available tasks.

### 4.3. Market-Based Algorithm

A market-based algorithm used in [9] for distributed sensing tasks was applied with application-specific modifications. In this approach, the bid of sensor *k* to task *i* is defined as (14):

(14) $$Bid_{ik}=F_{ik}+\delta \left(V_{ik}\frac{1}{D_{ik}}-F_{ik}\right)if\Delta_{i}>0$$

where *F_ik_* serves as the reservation price of task *i*, and *δ* is a control parameter with values between 0 and 1. A task *i* is selected by sensor *k* if it maximizes its bid value:

(15) $$Select=\max(Bid_{ik})$$

### 4.4. Greedy Algorithm

A greedy algorithm that was used previously for a multi target observation problem with broadcast messaging [10] was modified to fit the described problem. The greedy algorithm was set to perform sensor allocation based on the best possible allocation of each individual sensor to task that maximizes *V_ik_/D_ik_*, where *V_ik_* is the *k*-th sensor’s performance on the *i*-th task and *D_ik_* is the Euclidean distance between the sensor and the task:

(16) $$task_{i}=\underset{i\in Z}{\max}\left(\frac{V_{ik}}{D_{ik}}\right)\;\mathrm{if}\;\Delta_{i}>0$$

where *task_i_* is the task chosen by the *k*-th sensor, and *Z* is the set of tasks within *k*-th sensor range. Note that *Z* is a subset of all *M* available tasks.

### 4.5. Bees System

A BS used in [8] for a traveling salesman problem was applied with application-specific modifications. This algorithm uses an artificial colony of bees to optimally allocate sensors to tasks. When a new task is introduced, the algorithm is able to replace the obtained allocation with a new improved allocation:

(17) $$p_{ik}=\frac{e^{\rho F_{i}-\theta D_{ik}}}{\sum_{j=1}^{M}e^{\rho F_{j}-\theta D_{jk}}}if\Delta_{i}>0$$

where *ρ* and *θ* are control parameters that bias the importance of the priority and distance, respectively (*ρ*, *θ* > 0; *ρ*, *θ ϵ R*).

## 5. Simulation Setup and Analysis

In this research, three types of sensors are considered with different detection ranges and performances. Experiments were performed for a square-shaped simulation arena, but it can be easily extended to relatively complicated geometries. In addition, three deployment strategies have been considered as shown in Figure 2; (a) deterministic deployment (e.g., grid deployment); (b) random deployment, i.e., uniformly distributed random deployment; and, (c) random and biased deployment, i.e., normally distributed random deployment. Deployed sensors are marked as blue circles and tasks are marked by dots (black dots represent tasks that were completed, and red dots represent non-completed tasks). In these figures, there are 200 tasks (final distribution of tasks on the map) and 100 sensors distributed over the map. Effects of various sensor densities were examined.

The parameters that were used for the simulation are presented in Table 1. Tasks were uniformly distributed in the arena based on [2,63]. The sensors’ range values were predefined to cover 15 to 45 m relative to the size of the arena (100 × 100 m). An inherent noise in each sensor was introduced according to typical operational distance ranges of known commercial sensors [64] relative to the arena size: acoustic sensor, seismic sensor, and forward looking infrared radar (FLIR). The detection range of these sensors is given by:

(18) $$R_{seismic}=0.8\times R_{acoustic}=1.25\times R_{FLIR}$$

The numerical computations were performed on a PC with 2.00 GHz CPU, and 8GB of RAM, using Matlab R2012a software. The following performance measures were analyzed to compare the different allocation algorithms:

- System performance as defined in (1).
- Tasks completion time as defined in (9).
- Number of unallocated tasks is defined by:
  (19) $$\lambda =\sum_{i=1}^{M}task_{i}\;\mathrm{if}\;\sum_{k=1}^{N}V_{ik}=0$$
  where *λ* is the number of unallocated tasks, *M* is the total number of tasks in the system, *N* is the total number of sensors in the system, *i* is the index of current task and *V_ik_* is the *k*-th sensor’s performance on the *i*-th task.
- Number of tasks allocated to a sensor *k* is defined by:
  (20) $$\chi_{k}=\sum_{i=1}^{M}task_{i}\;\mathrm{if}\;V_{ik}>0$$
  where *χ_k_* is the number of treated tasks by the *k*-th sensor, *M* is the total number of tasks in the system, *i* is the index of current task and *V_ik_* is the *k*-th sensor’s performance on the *i*-th task.

The mean values that were obtained from 100 independent runs of MDBA, DBA, BS, market-based, and greedy algorithms, were compared at the statistical confidence level of 95%. The MDBA performance was simulated for three sensor distributions based on [65]: grid, uniform, and normal distributions.

In order to evaluate the system’s scalability, simulation analysis was conducted for different numbers of sensors and tasks. In the initial setup, 80 sensors were uniformly distributed in the area. Twenty additional sensors were introduced into the system in two phases. In the last phase, 10 sensors were removed.

The control parameters, *α*, *β*, and *γ* provide a mechanism to adjust the sensor swarm behavior. The values of *α*, *β,* and *γ* were changed in order to bias the resulting sensors’ allocation and to test the system’s performance for different values of these parameters: *α* = *β* = *γ* = 1, *α* = 2*β* = 2*γ*, *α* = 2*β* = *γ*, *α* = 2*β* = 0.5*γ*.

The range for these parameters was set according to [51,66]. The algorithm was able to converge in all of the scenarios.

## 6. Results

**Figure 3:**
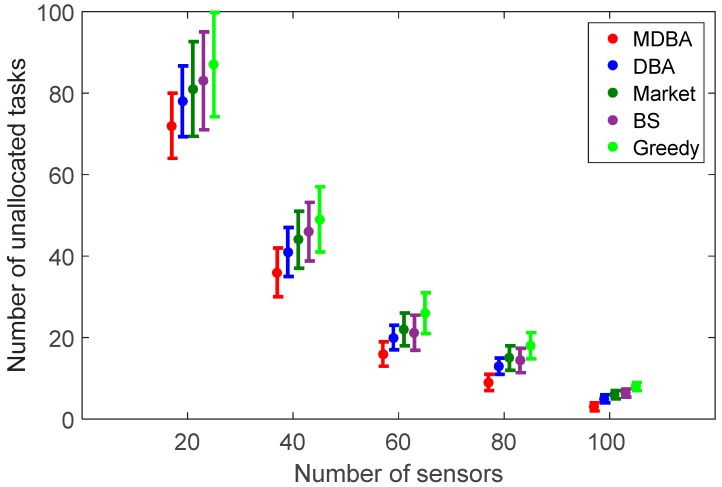
Number of unallocated tasks for a different number of deployed sensors for each algorithm with uniformly distributed random sensors deployment. The dots represent mean values of 100 independent runs and the bars represent standard deviation values.

Results revealed, as expected, that as the number of sensors increased, the mean number of unallocated tasks decreased, respectively, for all of the algorithms (from 72.53 to 4.74, from 78.25 to 5.88, from 81.14 to 6.07, from 83.13 to 5.98 and from 87.65 to 8.61 in MDBA, DBA, market-based, BS and greedy algorithms, respectively; Figure 3). By adding more sensors, the impact of their relative contribution decreases due to the overlapping range coverage (e.g., the difference in the mean number of unallocated tasks between 20 sensors to 40 sensors and between 80 to 100 sensors is 36 and 6 respectively). Therefore, optimal sensor density can be changed upon desired geometry of the arena and must be obtained through simulations. The bio-inspired algorithms MDBA and DBA resulted in a lower number of unallocated tasks than the market-based and greedy algorithms by 45.9% on average (from 29.31, std = 4.07 to 42.76, std = 5.66, *p* < 0.05) and with maximum of 81.7% difference (from 4.74, std = 0.97 to 8.61, std = 1.09, *p* < 0.05). The high number of unallocated tasks (72) when using 20 sensors was due to the limited coverage range of the sensors.

**Figure 4:**
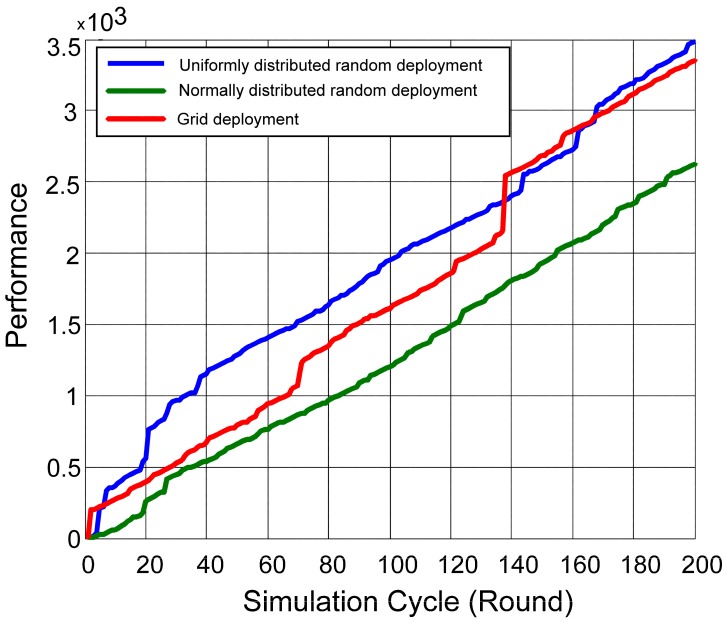
Modified Distributed Bees Algorithm (MDBA) Performance based on different sensor distributions.

The best performance of MDBA was obtained for sensors with a uniform distribution (Figure 4), resulting in the shortest task completion time with 5.1% and 26.3% improvement from the grid and normal distributions, respectively (*p* < 0.05). The normally distributed random sensor deployment achieved lower performance due to the fact that most of the sensors were deployed in the center of the arena. Thus, tasks that were generated in the edges of the arena were treated with lower performance. The performance changed as time advanced since sensors were allocated to the high priority tasks that were generated in a particular simulation cycle.

**Figure 5:**
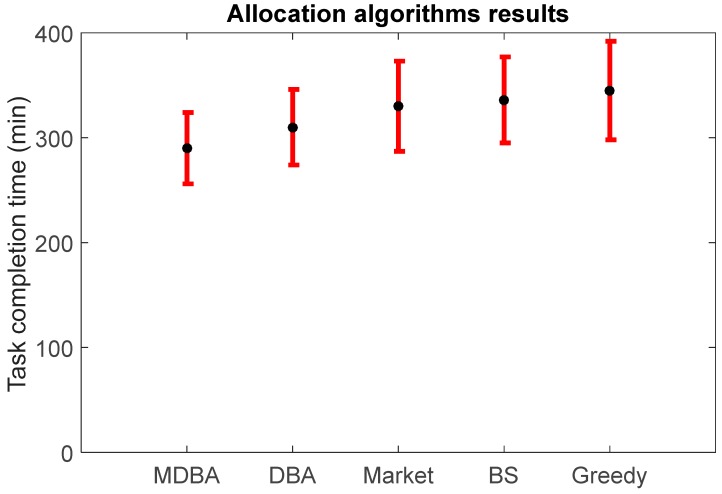
Average performance comparison of four sensor allocation algorithms. Black dots represent mean values of 100 independent runs and red bars represent standard deviation values.

The task completion times of 100 sensors using MDBA, DBA, market-based, BS and greedy algorithms are shown in Figure 5. The MDBA algorithm resulted in the lowest tasks’ completion time (291.21 min, std = 34.23, which is 6.6% lower than the second best algorithm, 310.12 min, std = 34.79, and 18.3% when compared to the greedy algorithm, 344.41 min, std = 35.21, all with *p* < 0.05).

**Figure 6:**
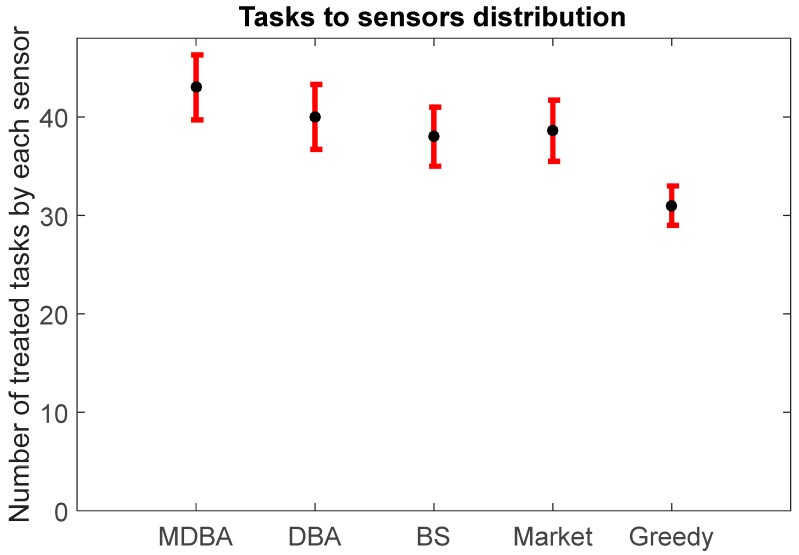
Average number of treated tasks by each sensor in four algorithms.

The greedy algorithm resulted in the longest completion time (346.36 min), due to its’ intrinsic greedy logic, which resulted in constant occupation of the best performing sensors without attempts of creating allocation combinations that optimize the overall performance. Also, it must be noted that despite the shorter completion time (291.21 min), MDBA resulted in a higher average number of tasks (43, std = 3.37) that were treated by each sensor when compared to the market-based algorithm (38, std = 3.25, with 329.34 min run time) and greedy algorithm (31, std = 3.12, with 346.36 min run time). In this sense, MDBA has a slight disadvantage in excessive sensor occupation (Figure 6), which can result in a higher failure rate and eventually reduce the overall system availability; however, these risks could be reduced by a more redundant sensor design. For example, if the system is designed with several redundant sensors in addition to the operational sensors, then the system’s availability will increase.

**Figure 7:**
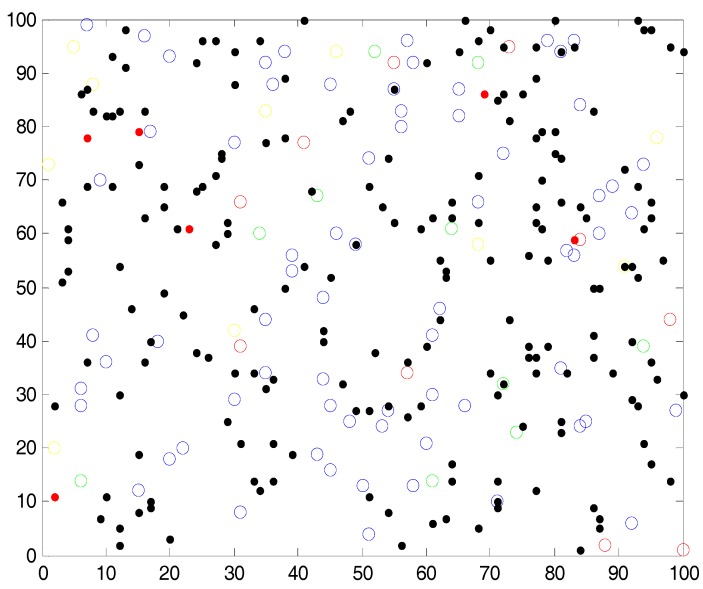
Simulation map of the arena with subsequently introduced sensors—initially deployed sensors are marked as blue circles, deployed sensors at phase 1 are marked as green circles, deployed sensors at phase 2 are marked as yellow circles, removed sensors at phase 3 are marked by red circles, incomplete tasks are marked by red dots and completed tasks are marked by black dots.

The system was able to scale-up with different numbers of sensors and tasks, and to maintain the ability to allocate sensors to the upcoming tasks (Figure 7). In all three phases that were simulated by addition and subtraction of sensors during the simulation, the system continued to allocate sensors and achieved an average value of tasks completion time of 312.55 min, std = 35.33.

**Figure 8:**
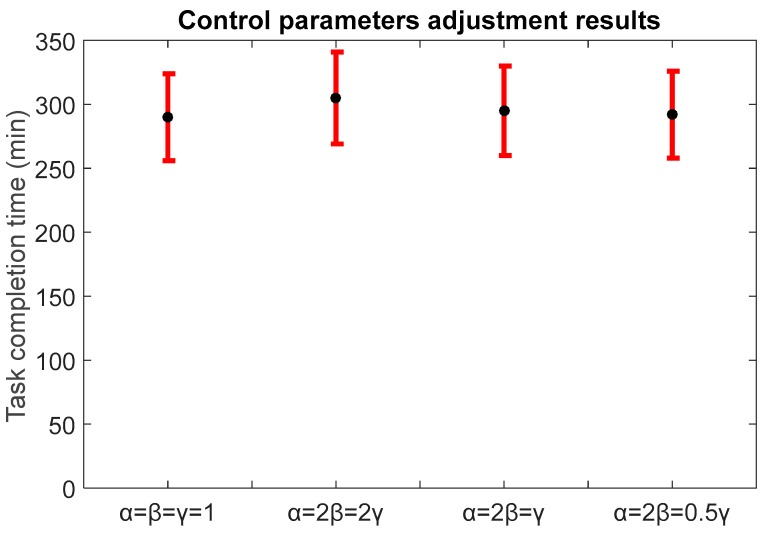
Average performance comparison of MDBA algorithm with different *α*, *β* and *γ* values. *α* = *β* = *γ* = 1; *α* = 2*β* = 2*γ*; *α* = 2*β* = *γ; α* = 2*β* = 0.5*γ*.

A simulation with different values of *α*, *β,* and *γ* was conducted and revealed that the setup of *α* = *β* = *γ* = 1 achieved the best results (291.21 min) in terms of task completion time (Figure 8), outperforming systems with *α* = 2*β* = 2*γ*, *α* = 2*β* = *γ* and *α* = 2*β* = 0.5*γ* by 5.2%, 1.8%, and 0.7%, respectively (*p* < 0.05). These parameters play an important role in defining system’s overall behavior. For example, they can be set to prioritize closer tasks and reduce traveled distances at the expense of the execution time (useful feature for mobile multi-sensor systems). Allocating the tasks according to their priorities would decrease the execution time of crucial tasks at the expense of longer execution of less important tasks.

## 7. Conclusions

The MDBA algorithm efficiently allocates a large group of heterogeneous sensors to upcoming tasks, which are unknown in their spatial and temporal distributions; it provides scalability in terms of the number of tasks and sensors. It resulted in statistically significant 7% better performance in terms of task completion time using 100 sensors in uniform distribution, with respect to the second-best algorithm, and 19% better with respect to greedy algorithm, which performed the worst. The control parameters, *α*, *β*, and *γ* provide a mechanism to adjust the sensor swarm behavior and bias importance of the priority, distance and sensors performance respectively. A simulation of these parameters revealed that the best task completion time was achieved by using *α* = *β* = *γ* = 1. Uniform distribution of the sensors resulted in better system performance with the shortest task completion time, with 5% and 26% improvement from the grid and normal distributions, respectively (*p* < 0.05).

Homogeneous agents can be treated as a sub-problem. In this case, MDBA and DBA will report the same results (see [48] for example, of DBA implementation to homogeneous agents).

The limitation of this work is that the proposed algorithm MDBA has a probabilistic nature. Due to its decentralized nature, MDBA cannot guarantee an optimal solution, but it can provide sub-optimal solutions to NP-hard problems; it yielded better results in comparison to other algorithms analyzed in this paper. The decentralized decision making process of the agents ensures system robustness and reliability in case of single sensor failure.

In real-world applications, communication delay between sensors must be considered because it can increase task completion time.

The proposed algorithm could be applied in many applications with distributed sensors, such as facility monitoring in industry, fire monitoring with in-field distributed sensors, surveillance, military and homeland security, among others. Furthermore, this work can be expanded to multi-agent and multi-robot applications such as search and rescue missions and path formation. Ongoing work is dealing with additional applications of the algorithm such as supply network security monitoring by a distributed sensor network and police officers tasks allocation optimization dealing with various crime incidents.

## Figures and Tables

**Table 1:** Summary of parameter values for simulation analysis.

Parameters	Values
Area dimensions	100 × 100 m
Number of sensors (‘bees’)	20, 40, 60, 80, 100
Number of tasks	200
Task completion time	Randomly distributed from 0 to 10 min
Simulation duration	200 steps
Tasks location	Uniformly distributed at random
Control parameters	*α* = *β* = *γ* = 1, *α* = 2*β* = 2*γ*, *α* = 2*β* = *γ, α* = 2*β* = 0.5*γ*
Sensors location	Uniformly distributed random
	Normally distributed random
	Grid deployment
Tasks arrival time	Every 1 step
Sensors range coverage	Predefined from 15 to 45 m
